# The importance of measuring psychosocial functioning in schizophrenia

**DOI:** 10.1186/1744-859X-10-18

**Published:** 2011-06-24

**Authors:** Sofia Brissos, Andrew Molodynski, Vasco Videira Dias, Maria Luísa Figueira

**Affiliations:** 1 Janssen-Cilag Pharmaceutical, Lisbon, Portugal; 2 Lisbon's Psychiatric Hospitalar Centre, Lisbon, Portugal; 3 Social Psychiatry Group, Oxford University Department of Psychiatry, Oxford, UK; 4 Santa Maria's University Hospital, Department of Psychiatry, Lisbon, Portugal

## Abstract

**Background:**

Schizophrenia is among the most disabling of mental illnesses and frequently causes impaired functioning. We explore issues of definition and terminology, and the relationship between social functioning, cognition, and psychopathology considering relevant research findings.

**Methods:**

The present article describes measures of social functioning and outlines their psychometric properties. It considers their usefulness in research and clinical settings. Treatment aims and objectives are explored in the context of cognitive and social functioning. Finally, we identify areas for developing research and refining the measurement of social functioning.

**Results:**

The definition and measurement of social functioning in schizophrenia remains a complex and disputed area. The relationships between symptoms, cognitive functioning and social functioning are complex but we are beginning to understand them better. Scales for measuring functioning in clinical practice must be brief and sensitive to change and the Personal and Social Performance (PSP) scale may offer several advantages in these regards. Brief cognitive assessments focusing upon the domains most commonly affected in schizophrenia, such as verbal memory and executive functions, should be coadministered with measures of functioning.

**Conclusions:**

The use of validated scales for schizophrenia that are sensitive to change over the course of the illness and its treatment, should allow for a better understanding of patients' functional disabilities, enabling better and more comprehensive monitoring and evaluation of both pharmacological and non-pharmacological treatment strategies.

## Background

Despite the most distinctive symptoms of schizophrenia being those such as delusions and hallucinations, functional deficits are a core feature of the disorder. In fact, diagnostic doubts often arise if a patient regains his/her previous level of functioning after a psychotic episode [1]. Decline in social functioning is one of the hallmarks of schizophrenia and may serve as a predictor of outcome.

The treatment of schizophrenia has evolved substantially in recent decades, with improvements in pharmacological interventions contributing to the deinstitutionalization of many patients. Second generation antipsychotics were introduced and generally had fewer side effects, especially regarding movement disorders. However, it is apparent that the isolated treatment of symptoms is not enough to reinstate good performance occupationally and in interpersonal relationships [2].

Pharmacotherapy and other interventions are expected to have a positive influence in a wider sense. Many clinicians hope for and expect improvements in social integration, professional skills, and the quality of interpersonal relationships following intervention [2]. Increasingly, symptom remission and ultimately recovery are advocated for as achievable treatment goals [3-5]. Alongside symptom remission, the goals of treatment must be to improve psychosocial functioning and quality of life through a variety of interventions [6].

This paper explores issues of definition and terminology and considers the relationship between social functioning, psychopathology and cognition.

### Psychosocial functioning in schizophrenia

Deficits in psychosocial functioning are a core feature of schizophrenia. They can be observed in its early stages, during acute exacerbations, and as part of the residual syndrome [7]. Such impairments include poor social interaction, difficulties in maintaining relationships with family and friends, and/or inadequate performance in the workplace [8].

Despite the recent widespread use of the term 'social functioning', there is limited consensus even about its definition. 'Social functioning' is often used interchangeably with a variety of similar and overlapping concepts, such as 'social performance', 'social adjustment' (how a person conforms to social expectations), 'social dysfunction' (an impaired ability to get along with others and function in society), 'social adaptation' (one's ability to live in accordance with interpersonal, social and cultural norms), and 'social competence' (the overall ability of a person to impact favourably on his or her social environment) [6].

There is no clear standard for levels of accomplishment in these functional domains in the general population, and attempting to do so with the mentally ill remains a challenge [9].

Social functioning has been defined globally as the capacity of a person to function in different societal roles such as homemaker, worker, student, spouse, family member or friend. The definition also takes account of an individuals' satisfaction with their ability to meet these roles, to take care of themselves, and the extent of their leisure and recreational activities [10].

The importance of social functioning in the assessment of patients with schizophrenia is acknowledged in the *Diagnostic and Statistical Manual*, fourth edition, text revision (DSM-IV-TR) [1] and it is stated that measurement of social functioning should be integral to the assessment of the effectiveness of antipsychotic drugs in schizophrenia.

### Sociodemographic factors and psychosocial functioning

The fact that younger patients have more difficulties in achieving functional remission may indicate that social deficits are present before the onset of psychotic symptoms [11]. Occupational status at admission has been shown to be predictive of functional outcome, as unemployed patients show significantly worse functional outcomes [11-13]. Patients with longer overall illness duration appear to have less favourable functional outcomes [11,14], as do patients with illnesses characterized by episodes of long duration [11].

### Psychopathology and psychosocial functioning

The early belief that an improvement in positive symptoms would automatically lead to improvements in multiple areas of daily living has now been empirically refuted [2].

Other symptoms may have more influence on psychosocial functioning than positive ones. Depressive symptoms negatively impact upon social functioning independently of other symptoms, predicting occupational and interpersonal performance [15]. Negative symptoms have also been identified as important determinants of psychosocial functioning in schizophrenia [16-19]. This would appear to 'make sense' but some studies have found that such negative symptoms were unrelated over time to scores on performance-based measures of functional capacity. This would seem to indicate that the relationship between negative symptoms and functional outcome is complex [19,20].

There is a high degree of intercorrelation between negative symptoms and cognitive deficits. It is therefore difficult to prove that neurocognition has a direct effect on functional outcome as the relationship is partially mediated by symptoms. A recent meta-analysis involving 6519 patients [18] found that, although neurocognition and negative symptoms are both predictors of functional outcome, the relationship between neurocognition and outcome might be at least partly mediated by negative symptoms. Suicidality in patients with schizophrenia is also predictive of a worse functional outcome [11].

### Cognition and psychosocial functioning

Cognitive deficits are a core feature of schizophrenia, and may be to some extent independent of other symptoms [21]. They may precede the onset of illness, becoming more pronounced in the prodrome and early years following diagnosis, and then settle into a stable pattern [22]. However, there is substantial interpatient heterogeneity, and even patients who perform within the normal range on neurocognitive testing are impaired relative to their estimated intellectual functioning [15].

Impairments are found across most domains; attention, working memory, verbal fluency, processing speed, executive functions, and verbal memory. There may also be superimposed severe deficits in domains such as verbal learning and executive function [23].

The importance of cognition in schizophrenia hinges on its relationship to real-world functioning [24]. Cognitive deficits have been shown to be linked to impairment in functional status among patients with schizophrenia in both cross-sectional [25-27] and longitudinal studies [27-29]. Furthermore, studies of those in supported employment affirm the close relationship between cognitive and professional skills [30].

Verbal memory has been proposed to be one of the main predictors of psychosocial functioning, being independent of gender [27]. This supports the hypothesis that cognitive variables are better predictors of functioning than symptomatology. However, a longitudinal 7-year follow-up study of patients after their first episode of illness showed that cognition appeared to explain less of the variance in outcome, which was also mediated by negative symptoms [17].

Certain cognitive abilities appear particularly important for the acquisition of social or living skills, while others may be important for the deployment of these skills in real time in the real world [31].

Findings from longitudinal studies provide initial support for the hypothesis that changes in neurocognitive ability are associated with changes in functional status among patients with schizophrenia [28]. However, there seems to be a possible 'threshold' relationship between cognitive and functional status whereby improvement in cognition may have to reach a certain level before a meaningful change in functional status occurs [28]. If this threshold hypothesis is supported by future research, it would suggest that the treatment of cognitive impairment is a critical step towards helping patients with schizophrenia to improve in meaningful functional domains [28]. Cognitive remediation might then be viewed as an initial and critical step in promoting functional recovery [31].

Social cognition has been suggested as an important mediating variable in the relationship between neurocognition and functional outcome. Neurocognition affects social cognition. Poorer social cognition leads to social discomfort on the job. This in turn leads to poorer rehabilitation outcomes [32].

Emotional experience also appears to be an important determinant of functional outcome in schizophrenia and one that is independent of neurocognition and social cognition [33]. In stabilized community patients with schizophrenia, affect recognition deficits have significant consequences for social functioning, again independently of basic neurocognition [34].

Existing measures of functional assessment do not adequately address the relationship between cognitive impairment and function. Although measures of practical cognition are relatively objective, efficient, and readily standardized, they may not be closely related to a patient's actual functioning in the community [35].

This is central to future clinical trials of cognitive enhancing strategies and outcome measures that are specifically designed to be responsive to change in cognition should be developed [28].

Due to their close relationship, it is important that appropriate tests of functioning and cognition are coadministered [36]. A substantial proportion of the variance in several different neuropsychological and functional outcomes can probably be measured by a small number of easy to complete neuropsychological tests. Since occupational functioning is known to be strongly associated with verbal memory and executive functions [23], these domains should be addressed when testing the relationship between cognition and function in patients with schizophrenia.

The Measurement and Treatment Research to Improve Cognition in Schizophrenia (MATRICS) Project produced a battery of tests, the MATRICS Consensus Cognitive Battery (MCCB), designed to assess cognitive treatment effects in clinical trials of patients with schizophrenia [37]. In validation studies, and in antipsychotic trials of stable patients, the MCCB demonstrated excellent reliability, minimal practice effects and significant correlations with measures of functional capacity [37]. Recently Shamsi *et al. *[19] found significant relationships between scores on the MATRICS cognition battery, negative symptoms and aspects of functional outcome in 185 stable schizophrenia patients. Work or educational functioning was predicted by working memory performance and negative symptoms, residential status (independent living) was predicted by verbal memory scores, and social functioning was predicted by social cognition, attention and negative symptoms.

The Brief Assessment of Cognition in Schizophrenia (BACS) assesses the aspects of cognition found to be most impaired and most strongly correlated with outcome in patients with schizophrenia [38]. It requires about 30 min to complete, has high reliability, and was found to be as sensitive as a standard battery of tests that required over 2 h to administer, making it a promising tool for assessing cognition in clinical trials. Other brief assessments such as the Screen for Cognitive Impairment (SCIP) also show adequate validity as a screening tool for cognitive deficit in both schizophrenia and bipolar patients [39]. Other simple to use tasks such as the digit symbol coding, which is reliable and easy to administer, and taps an information processing inefficiency that is a central feature of the cognitive deficit in schizophrenia [40], can easily be used in clinical settings.

Further research is needed to determine whether in clinical practice responses to pharmacological and remediation treatments can be captured with brief assessments in a meaningful way [41].

### Measurement of social functioning

Despite the fact that impaired social functioning has historically been considered an important characteristic of schizophrenia, the assessment of personal and social functioning remains a relatively undeveloped area of some controversy and uncertainty [42,43]. A range of different instruments to assess social functioning is available (for a recent review see Figueira and Brissos [43]), but there is still no real agreement on which scale to use for which purpose.

The assessment of real-world functioning presents complex challenges from variability in the operational definition of functional outcome, to problems in identifying optimum information sources [42]. Judging an individual's functional recovery can be a difficult task for health care professionals [44].

To enhance the measurement of outcomes in social, residential, and vocational domains, the VALERO Expert Survey selected 6 out of 59 nominated measures [42]. The two social functioning measures with the highest ratings by the experts were the Social Functioning Scale (SFS) and the Social Behavior Schedule (SBS). The SBS takes 15 min to be rated by an informant, assessing the past month's functioning in 21 areas. The SFS is an informant report completed by the patient or a relative, but it has 79 items. Both may well be too lengthy for routine clinical use, a common issue with social functioning measures.

There are several limitations with the current measurement of social functioning, and most scales were not developed for use in schizophrenia. There remains a pressing need to develop appropriate measures for this population that will capture the unique clinical features of the disorder as well as the impact of our interventions upon it [6].

There is often poor assessment of the psychometric properties of those scales that are in use, with little evidence of their validity, reliability, responsiveness and sensitivity in schizophrenia [6]. Measures of social functioning need to be sensitive to small changes in behaviour, as many patients have long-term and severe handicaps that are slow to change. Relatively minor behavioural changes can lead to significant shifts in social functioning and acceptance over time [6].

A major issue remains the lack of consensus concerning the definition and evaluation of social functioning. This in part appears to be related to the lack of distinction between objective (that is, employment, presence of a significant other, independent living, and social contacts) and subjective indicators (that is, the patient's ratings of their feelings, thoughts and views concerning their social situation) [7,10].

Many instruments have been developed to assess community functioning, but overall insufficient attention has been paid to psychometric issues and many instruments are not suitable for use in clinical trials [45]. Consumer self-report, informant report, ratings by clinicians and trained raters, and behavioural assessment all can provide useful and valid information in some circumstances and may be practical for use in clinical trials. A major limiting factor in the development of instruments appears to have been a failure or inability to develop a suitable model of functioning and its primary mediators and moderators [45].

Several external factors are also likely to affect models of functional outcome, particularly at the post-competence level. For example, social stigma, lack of social support, and financial resources might well be barriers to real-world functioning even when skill competence is improved [45].

Recently Burns and Patrick [6] reviewed the current use of social functioning scales both in the assessment of schizophrenia and as outcome measures in trials of antipsychotic agents. Complex instruments are available to measure psychosocial functioning but by their very nature are usually detailed and time consuming. They tend to require detailed knowledge of the patient and his/her actual circumstances, staff training, and an extended interview [2]. As a result such instruments are not readily usable in day-to-day practice and simpler measures of functioning are required.

Being quick and simple to use in either research or clinical practice, the Global Assessment of Functioning (GAF) scale has been the most used measure of social functioning [46]. However, the GAF's single score includes symptoms and these can influence the rating, making it a less 'pure' measure of functioning. Studies have shown several problems with the GAF, for example concerning its validity and reliability, and guidelines for rating the GAF are not comprehensive [47]. The Social and Occupational Functioning Assessment Scale (SOFAS) [1] was developed in an attempt to eliminate this difficulty. It is a very general instrument and does not include clear operational instructions for rating the severity of disability.

Morosini *et al. *[48] developed the Personal and Social Performance (PSP) scale from the SOFAS. Ratings are based on the assessment of four (theoretically) objective indicators: (1) socially useful activities, including work and study; (2) personal and social relationships; (3) self-care; and (4) disturbing and aggressive behaviours, rated on a six-point severity scale. The interviewer assigns a global score based upon interview information regarding the four main areas discussed and any additional information obtained that aids in making a clinical judgment. Thus, the assigned score is not simply a composite of the four items [48,49] but allows for the tracking of functioning in the four domains over time and in different phases of the illness. It is quick to use, often only taking a few minutes. It has been used in randomized controlled trials and has been proposed as being particularly well suited to the role of assessing outcome in antipsychotic trials [6]. It has been validated in several countries [7,50-53], in both acute and stabilized patients, overall demonstrating good reliability, validity and sensitivity to change over time.

More recently, the Schizophrenia Outcomes Functioning Interview (SOFI) was developed to measure community functioning related to cognitive impairment and psychopathology [54]. It has demonstrated good reliability and construct validity and captures more comprehensively the functioning of patients in the real world as compared to other performance-based (proxy) measures [54].

Performance-based measures of the ability to perform social and everyday living skills, such as the University of California, San Diego (UCSD) Performance-Based Skills Assessment (UPSA), are becoming more widely used to assess functional capacity in this group [44]. They are also being used as outcome measures in pharmacological and cognitive remediation studies in schizophrenia. They may be most effective in predicting independent living and work but are usually time consuming and require special resources.

It will be apparent from the previous section that no 'gold standard' measure has been developed to date. The development and evaluation of further scales to assess functioning in schizophrenia is a pressing need.

### Limitations of functioning measures

Most scales have been developed in Western societies. They may not generalize well to other cultures as the definition of functional recovery differs with individual and cultural factors [44]. Outcomes may be influenced by economic and political factors, particularly in the current global financial crisis.

Many assessment measures have been developed for particular research projects and are lengthy and impractical for use in clinical settings [55].

Self-report measures have the potential to give greater insight but have inherent biases. Patients with schizophrenia may have only partial insight into their illness, limiting the reliability of using self-report measurements [56]. However, ratings made by others may be limited by poor knowledge about the patient's day-to-day life. This is common among clinicians who see patients for only brief office visits [55]. Family members have been proposed as alternative raters of patient functioning, and are often excellent sources of information [55]. However, not all patients maintain regular contact with their families and independent raters are too costly an addition to the assessment process.

Rating scales developed for the general population or even for less severely ill patients may demonstrate 'floor' and/or 'ceiling' effects in this population [55]. In the former the functioning of persons with serious mental illness may fall at the bottom of a scale with a lack of discrimination at these lower levels. Ceiling effects are less likely but again lead to a lack of discrimination, this time at the upper end of a scale.

### Aims of treatment

Improved personal and social functioning has become an important outcome measure in randomized controlled trials of antipsychotics and innovative psychosocial therapies [6,57]. It is important that routine clinical data gathering or research in this area should assess objective and subjective indicators of broad social functioning. This will enable us to increase our knowledge regarding such outcomes in routine care and with novel interventions, while capturing the views and experiences of the patients concerned [10].

Although several psychosocial interventions have been shown to improve personal and social performance [58,59], pharmacotherapy trials have often neglected to measure these outcomes. Despite the steady increase over the last two decades in the number of clinical trials reporting social functioning as an outcome measure in schizophrenia, only a few controlled trials of antipsychotic drugs have done so. The majority of randomized, controlled trials were of short duration (6-12 weeks), which is almost certainly not long enough to meaningfully assess change in social functioning in this group.

A recent study concluded that even modest gains in cognitive performance with second-generation antipsychotic treatment account for significant improvements in performance-based social skills [60]. The authors concluded, however, that cognitive performance was less responsive than social competence. Longer-term trials incorporating broad efforts to reduce cognitive dysfunction, cultivate and encourage the deployment of skills, and reduce negative and depressive symptoms may demonstrate a reduction in disability. If this was found to be the case, it would be of great importance.

In developed health care systems and economies, demand for outcome data from managed care providers, consumer organizations, and state agencies is increasing steadily. This data is required to inform decisions about resource allocation, evaluate the effectiveness of interventions, and to measure the effects of change in the health care system [55]. It is important that measures introduced are those with an evidence base to support their clinical usefulness as well as their bureaucratic expediency. Failure to ensure this would represent a missed opportunity at a time of great change in many health care systems around the world.

## Conclusions

The recent upsurge in interest regarding social outcomes in schizophrenia is exciting and timely. Social functioning must be considered a crucial outcome measure in randomized controlled drug trials and in studies of innovative psychosocial therapies and service models.

Symptoms and cognitive deficits are known to impact on the social functioning of patients with schizophrenia. Since negative and depressive symptoms might be rate-limiting factors even with cognitive and functional skill attainment, new measures of social functioning need to be carefully designed and evaluated to avoid some of the pitfalls of earlier measures.

Inevitably, due to the complexity of the issues involved, most measures of social functioning in patients with schizophrenia have limitations. The most pressing need appears to be to develop and promote scales that are able to assess functioning independently of symptoms and which are feasible to use in both research and clinical settings. Brief cognitive assessments that focus upon the domains most commonly affected in schizophrenia, such as verbal memory and executive functions, can help us to determine response to pharmacological and other treatments, and should be coadministered with functioning measures.

In clinical practice such measures should be used prior to treatment to aid the development of a tailored intervention plan, and then during treatment and at its conclusion. This would enable us to robustly assess change in functioning levels with our interventions and would provide potentially useful data for healthcare planners and providers.

As clinicians know very well, real-world performance is the product of a complex array of abilities, deficits, and symptoms. Other factors such as social and cultural influences are involved and we need to be mindful of this when planning interventions. The use of validated scales for patients with schizophrenia that are sensitive to change over the course of the illness and of its treatment, will allow a better understanding of patients' functional disabilities, enabling better and more comprehensive monitoring of both pharmacological and non-pharmacological treatment strategies. This may lead in time to interventions that are increasingly focused on specific aspects of social functioning with the possibility of improved outcome as a result.

## Competing interests

SB is a psychiatrist and has been Medical Affairs Manager for Janssen-Cilag Portugal since April 2010. AM is a consultant psychiatrist in Oxfordshire affiliated to the Social Psychiatry Group in the Oxford University Department of Psychiatry. VVD is a clinical neuropsychologist affiliated to Santa Maria's University Hospital. He is a consultant for Angelini Pharmaceutical Portugal, and has received educational grants from Lundbeck, Sanofi-Aventis, Janssen-Cilag and AstraZeneca. MLF is a full professor of Psychiatry and Head of the Department of Psychiatry at Santa Maria's University Hospital.

## Authors' contributions

SB managed the literature search, and wrote the first draft of the manuscript. The data were analysed by SB, VVD, AM and MLF, who wrote the final draft of the manuscript. All authors contributed to and approved the final version of the manuscript.
